# Breast Remote Reading: Widely Desired But Home Workstations Show No Association With Job Satisfaction Or Burnout

**DOI:** 10.1093/jbi/wbaf039

**Published:** 2025-11-03

**Authors:** Ria Dawar, Lars J Grimm, Emily B Sonnenblick, Brian N Dontchos, Kristen Coffey, Sally Goudreau, Beatriu Reig, Sarah A Jacobs, Zeeshan Shah, Lisa Mullen, Vandana Dialani, Reema Dawar, James Sayre, Katerina Dodelzon, Jay R Parikh, Hannah S Milch

**Affiliations:** Department of Radiology, David Geffen School of Medicine at UCLA, Los Angeles, CA, USA; Department of Radiology, Duke University Medical Center, Durham, NC, USA; Department of Radiology, Icahn School of Medicine at Mount Sinai, New York, NY, USA; Department of Radiology, University of Washington, Seattle, WA, USA; Department of Radiology, Weill Cornell at New York-Presbyterian, New York, NY, USA; Depatment of Radiology, University of Texas Southwestern Medical School, Dallas, TX, USA; Department of Radiology, New York University Grossman School of Medicine, New York, NY, USA; Allina Health, New Ulm Medical Center, New Ulm, MN, USA; Elite Breast Imaging/Radiology Partners, El Segundo, CA, USA; Department of Radiology and Radiological Science, Johns Hopkins University School of Medicine, Baltimore, MD, USA; Department of Radiology, Beth Israel Lahey Health, Boston, MA, USA; Fu Foundation School of Engineering and Applied Science, Columbia University, New York, NY, USA; Department of Radiology, David Geffen School of Medicine at UCLA, Los Angeles, CA, USA; Department of Radiology, Weill Cornell at New York-Presbyterian, New York, NY, USA; Division of Diagnostic Imaging, University of Texas MD Anderson Cancer Center, Houston, TX, USA; Department of Radiology, David Geffen School of Medicine at UCLA, Los Angeles, CA, USA

**Keywords:** breast radiology, burnout, home workstations, job satisfaction, radiology remote reading

## Abstract

**Objective:**

Understand radiologists’ opinions regarding remote breast imaging and determine whether having home workstations is associated with greater job satisfaction or less burnout.

**Methods:**

A 43-question survey on remote breast imaging was distributed to Society of Breast Imaging members (July 6 to August 2, 2023). Questions regarding job satisfaction and burnout were included. Pearson’s chi-squared tests compared demographic variables and responses. Multiple-variable logistic regression assessed associations between home workstations and job satisfaction or burnout.

**Results:**

In total, 424 surveys were completed (response rate 13%, 424/3244). Among the third (31%, 132/424) of breast imaging radiologists with home workstations, top motivations included flexibility/work-life balance (67%; 88/132) and decreased commute time (51%, 67/132). Most felt that working from home improved their efficiency (65%, 86/132). Perceived drawbacks among all breast imaging radiologists included the inability to perform US or physical examination (71%, 300/424) and impaired patient contact (47%, 198/424). Most (57%, 240/424) wished for more breast imaging remote reading opportunities, and one-third (32%, 136/424) saw themselves in a 100% remote reading practice in the future. The majority (60%, 228/388) felt that remote reading would majorly or moderately improve radiologist wellness, but no significant association was found between having home workstations and job satisfaction (*P* = .301) or burnout (*P* = .140).

**Conclusion:**

The majority of breast imaging radiologists want more opportunities to work remotely, perceiving that it improves work-life balance and efficiency, albeit at the expense of patient contact. However, those currently working from home did not have higher job satisfaction or lower burnout.

Key Messages

- Of the one-third of surveyed breast imaging radiologists with home workstations, top motivations for reading from home included greater efficiency, improved flexibility/work-life balance, and decreased time commuting.
- The most common perceived drawbacks to remote breast imaging included the inability to personally perform breast US or physical examination and impaired patient contact.
- The majority of surveyed breast imaging radiologists wished that their practice provided more remote reading opportunities, and one-third could see themselves in a 100% remote practice in the future.
- Despite the perceived wellness benefits of remote work, breast imaging radiologists with home workstations did not exhibit greater job satisfaction or less burnout when compared with radiologists without home workstations.

## Introduction

Accelerated by the COVID-19 pandemic, remote work has dramatically increased in the United States over the past several years across diverse fields, even those previously thought to require an on-site presence.^1,2^ Practices, especially in high-demand fields such as breast imaging, are now incentivized to provide flexible working conditions to attract top talent.^3,4^ A 2022 nationwide survey demonstrated that 76% of breast imaging radiologists perceive that remote work improves work-life balance and reduces burnout.^5^ However, working remotely can present its own stress, such as an isolated work environment and home distractions.^6,7^ Thus, despite the perceived benefits of remote work, it is unknown whether breast imaging radiologists working remotely actually experience less burnout compared with radiologists working in-person.

Breast imaging has traditionally been seen as the face of the American College of Radiology Imaging 3.0 culture change as an in-person specialty.^8,9^ Thus, transitioning to remote breast imaging has been met with some controversy.^5^ No national guidelines exist to appropriately tailor remote workflow to allow for greater radiologist flexibility and patient access without compromising patient care. If remote breast imaging is to become a growing phenomenon, more study is needed to explore effective strategies to work remotely to benefit both radiologists and patients.

From a 2023 nationwide survey of Society of Breast Imaging (SBI) members, we previously reported on current practice patterns and planned future directions in remote breast imaging.^10^ We found that one-third of breast imaging radiologists read studies from a home workstation for a median of 25% of their clinical time. Half of these radiologists with home workstations read diagnostic breast imaging (mammograms and/or US) from home. Furthermore, three-quarters of breast imaging radiologists felt that remote breast imaging would be a significant practice pattern in the future.

Here we present additional data and analysis from this 2023 survey focusing on radiologist preferences and perceptions regarding remote breast imaging. We also examine whether the subset of breast imaging radiologists with home workstations demonstrates differences in reported burnout and job satisfaction. We hypothesized that breast imaging radiologists were seeking greater remote work opportunities and that those with home workstations would have less burnout and greater job satisfaction. These results may inform practices across the country as their remote reading workflows and opportunities evolve.

## Methods

This study received a waiver from the institutional review board at University of California - Los Angeles. A cross-sectional survey was designed to assess breast imaging radiologists’ experiences and feelings toward remote reading in the United States. In addition, 2 single-item validated questions on job satisfaction and burnout were included to assess whether the subset of respondents with home workstations had improved job satisfaction and/or less burnout.^11,12^ The full survey consisted of 43 multiple-choice questions, with a limited number allowing for multiple answers as well as free-response answer choices (Appendix A). Some questions specifically asked about home workstations, wherein “reading from home” was defined as reading “remotely from home or other personal remote site (eg, remote office space)” to account for participants who may travel to a personal office space. Other questions asked more generally about “breast remote reading,” which was defined as encompassing all types of remote breast imaging, including reading examinations from home and/or performing remote diagnostic visits either from home or a central/local site. Five questions were only answerable by respondents who read breast imaging from home. Sixteen questions—including 5 open-ended questions—were answerable only by respondents with personal experience or knowledge regarding remote diagnostic breast imaging.

The survey was developed by the SBI Patient Care and Delivery Committee, which included 12 fellowship-trained breast imaging radiologists and a mammography technologist. Prior to implementation, the survey was piloted at 8 breast imaging practices. Changes to the survey length, wording, and organization were incorporated based on this pilot testing.^10^ Results from objective questions about current use and planned future practice patterns of remote breast imaging were previously reported.^10^ This manuscript focuses on the subjective survey questions specific to breast imaging radiologist preferences and perceived benefits and drawbacks to remote breast imaging and home workstations as well as associations with job satisfaction and burnout.

The survey was created in SurveyMonkey (SurveyMonkey, Inc, San Mateo, CA) and distributed by electronic mail to the 3244 active physician members of the SBI practicing in the United States.^13^ The survey was open from July 6, 2023, to August 2, 2023. Completion of the survey was optional, and partial surveys could be submitted. Participants received no compensation. Survey responses were summarized descriptively using the SurveyMonkey software.^13^ Multiple-variable logistic regression analysis was used to assess relationships between having breast home workstations and job satisfaction as well as burnout. *P*-values < .05 were considered statistically significant. There were no controls for multiple-hypothesis testing.

Analyses were performed via Google Sheets (Google, Inc, Mountain View, CA) and Google Colaboratory (Google, Inc, Mountain View, CA).^14,15^ IBM SPSS Statistics software for Windows, version 28.0 (Armonk, NY), was used to perform the multiple logistic regression analysis.^16^

## Results

### Participant demographics

A total of 424 surveys were completed by the SBI physician members, who reported spending on average 87% of their clinical time on breast imaging. The overall response rate was 13% (424/3244). Mean age was 52 ± 12 years, and 73% (309/424) of respondents were women (Table 1). Most respondents identified as White (69%, 292/424), followed by 15% (63/424) as Asian American, 4% (15/424) as Hispanic, and 2% (8/424) as Black, with 10% (44/424) nonresponders. On average, respondents reported 19 ± 12 years of practice across a wide range of 44 states and all geographic regions. Practice group types included primarily private (45%, 191/424) and academic (24%, 103/424) with 22% (92/424) community practice affiliated with an academic medical center. Over half of respondents (57%, 240/424) reported having dependents at home. Demographic variables were similar between respondents with and without home workstations.

**Table 1.** Respondent and Breast Imaging Practice Characteristics

Characteristics	Total *N* = 424, *n* (%)	Home workstation *N* = 132, *n* (%)^a^	No home workstation *N* = 283, *n* (%)^a^
Age, mean ± SD (years)	52 ± 12	53 ± 11	51 ± 12
Years of practice, mean ± SD (years)	19 ± 12	20 ± 12	18 ± 12
Gender			
Woman	309 (73)	103 (78)	201 (71)
Man	98 (23)	26 (20)	72 (25)
Nonbinary	1 (0.2)	0 (0)	1 (0.4)
Did not respond	16 (4)	4 (3)	9 (3)
Race			
White	292 (69)	93 (70)	196 (69)
Asian American	63 (15)	18 (14)	45 (16)
Hispanic/Latino	15 (4)	8 (6)	7 (2)
Black/African American	8 (2)	3 (2)	5 (2)
Native Hawaiian or Pacific Islander	1 (0.2)	0 (0)	1 (0.4)
Other	1 (0.2)	0 (0)	1 (0.4)
Did not respond	44 (10)	11 (8)	28 (10)
Type of practice			
Academic	103 (24)	23 (17)	78 (28)
Private	191 (45)	56 (42)	133 (47)
Academic/private hybrid	92 (22)	30 (23)	61 (22)
Military/VA	3 (0.7)	1 (0.8)	2 (0.7)
Teleradiology	17 (4)	17 (13)	0 (0)
Other	14 (3)	5 (4)	8 (3)
Did not respond	4 (0.9)	1 (0.8)	1 (0.4)
Leadership role			
Yes	207 (49)	67 (51)	138 (49)
No	212 (50)	65 (49)	144 (51)
Did not respond	5 (1)	1 (0.8)	1 (0.4)
Dependents			
Yes	240 (57)	80 (61)	158 (56)
No	178 (42)	52 (39)	125 (44)
Did not respond	6 (1)	1 (0.8)	0 (0)
Region of practice			
Northeast	111 (26)	42 (32)	68 (24)
South	109 (26)	33 (25)	76 (27)
West	68 (16)	12 (9)	55 (19)
Midwest	64 (15)	18 (14)	46 (16)
Southwest	49 (12)	21 (16)	28 (10)
Did not respond	23 (5)	7 (5)	10 (4)

Abbreviation: VA, U.S. Department of Veterans Affairs.

^a^These 2 columns do not add up to the overall total of 424 survey respondents because 9 people skipped the question about whether they had home workstations. Also note each individual row might be slightly less than the total because of the small number (<3%) who skipped a given demographics question.

### Motivations and perceived efficiency of breast imaging radiologists with home workstations

Approximately one-third of respondents (31%, 132/424) read breast imaging examinations from home as part of their routine clinical practice and for a median of 25% of their breast clinical time (Table 2). These respondents reported spending an average of 90% of their clinical time overall on breast imaging. Reading breast imaging from home consisted of screening and/or diagnostic studies.^10^

**Table 2.** Motivations and Perceived Efficiency of Breast Radiologists With Home Workstations

Questions	Answers	*N* = 424	%
Do you read breast imaging exams remotely from home or other personal remote site (eg, remote office space) as part of your routine clinical practice?	Yes	132	31
	No	283	67
	Did not respond	9	2
Subset of people who said yes		***N* = 132**	%
What are your top 3 motivations for reading from home or a personal remote site?	Personal health issue	6	5
	Decrease time commuting	67	51
	Dependent care (children, elderly, etc)	30	23
	Personal preference working solo	13	10
	Flexibility/work-life balance	88	67
	Extra income	32	24
	Limit time spent discussing results/recommendations/procedures with patients	5	4
	Limit challenging patient interactions	3	2
	Other	42	32
	Did not respond	0	0
How does working from home or a personal remote site affect your efficiency?	Much less efficient	2	2
	Less efficient	17	13
	No change	25	19
	More efficient	44	33
	Much more efficient	42	32
	Did not respond	2	2

Top motivations for reading from home were flexibility/work-life balance (67%, 88/132), decreased time commuting (51%, 67/132), extra income (24%, 32/132), and dependent care (23%, 30/132) (Table 2). One-third (32%, 42/132) listed “other” motivations, and some of the free-response reasons included geographic restraints, greater efficiency, and practice requirements.

Reading from home to care for dependents was more common among women (28%, [28/101] vs men 4% [1/25]; *P* = .024) and younger breast imaging radiologists (72% [13/18] for ages ≤40 years vs 0%-24% for ages >40 years; *P* <.001) (Appendices B and C). Reading from home to increase flexibility/work-life balance was more common among radiologists with dependents (76% [59/78] vs 54% [27/50]; *P* = .019) and younger radiologists (ages ≤40 years: 89% [16/18]; ages 41-50 years: 79% [30/38]; ages >50 years: 22%-67%; *P* = .001) (Appendices C and D). Finally, the extra income from reading from home was more commonly cited by men (48% [12/25] vs 20% [20/101] women; *P* = .008). No significant differences were found in motivations to read from home in terms of region (*P* = .361) or practice type (*P* = .762) (Appendices E and F).

The majority (65%, 86/132) of respondents felt that working from home improved their efficiency with no significant differences based on gender (*P* = .503), practice type (*P* = .899), geographic region (*P* = .936), age group (*P* = .246), or having a dependent (*P* = .165).

### Perceived advantages and drawbacks of remote reading

**Figure 1.**
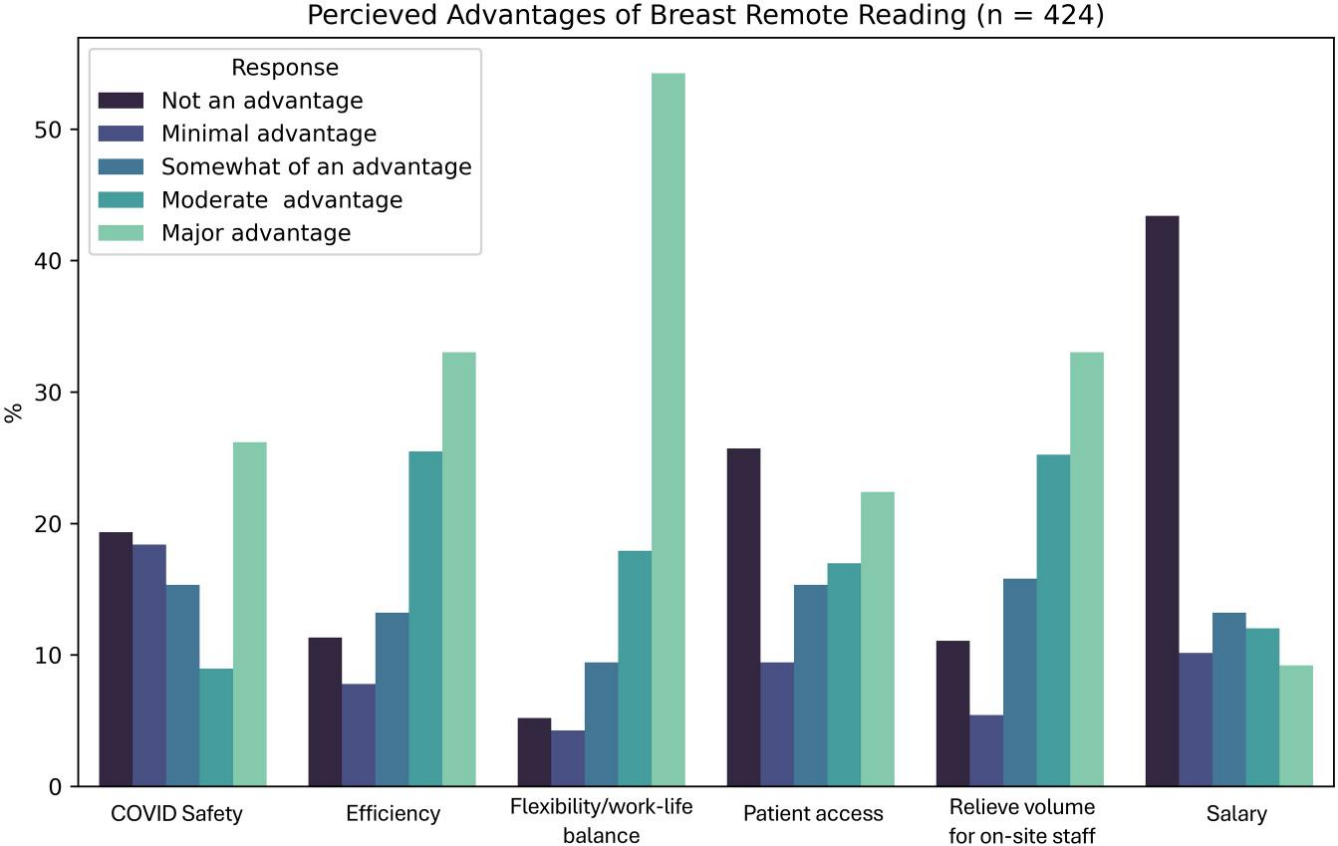
Responses to survey question 20: What do you see as the primary advantages of breast remote reading? Responses based on 5-point Likert scale ranging from “not an advantage” to “major advantage” (n = 424). Created from *SurveyMonkey*.^13^

All participants (regardless of whether they had home workstations) were asked to evaluate potential benefits of breast remote reading by ranking them on a 6-point scale from “not an advantage” to “major advantage.” The majority indicated that flexibility/work-life balance (72%, 306/424), efficiency (58%, 248/424), and relief of volume for on-site staff (58%, 247/424) were major or moderate advantages (Figure 1). Notably, 43% (184/424) of respondents stated that salary was not an advantage of remote reading.

**Figure 2.**
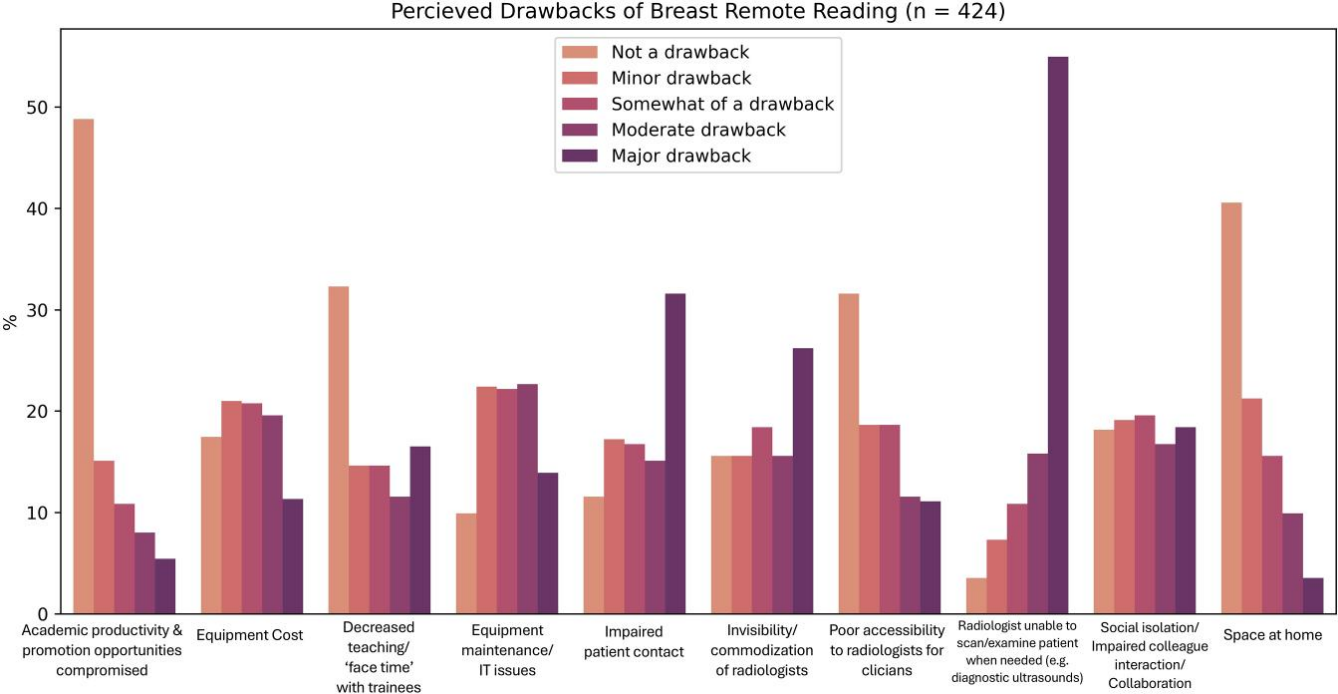
Responses to survey question 21: What do you see as the drawbacks of breast remote reading? Responses based on 5-point Likert scale ranging from “not a drawback” to “major drawback” (n = 424). Created from *SurveyMonkey*.^13^

Similarly, participants were asked to evaluate potential drawbacks of remote reading. Most participants felt that the inability to personally perform breast US or examine patients when needed (71%, 300/424) was a major or moderate drawback of remote reading (Figure 2). Impaired patient contact (47%, 198/424), invisibility/commoditization of radiologists (42%, 177/424), and information technology (IT) issues (37%, 155/424) were reported as other major or moderate drawbacks. For those who answered “other” in response to this question, some free-text answers indicated that while remote diagnostics were viewed as “bad,” reading screening examinations from home was considered “good.” Others noted that in-person patient interactions might reduce the likelihood of lawsuits and that remote work places a greater burden on on-site breast imaging radiologists for staff teaching and feedback.

**Figure 3.**
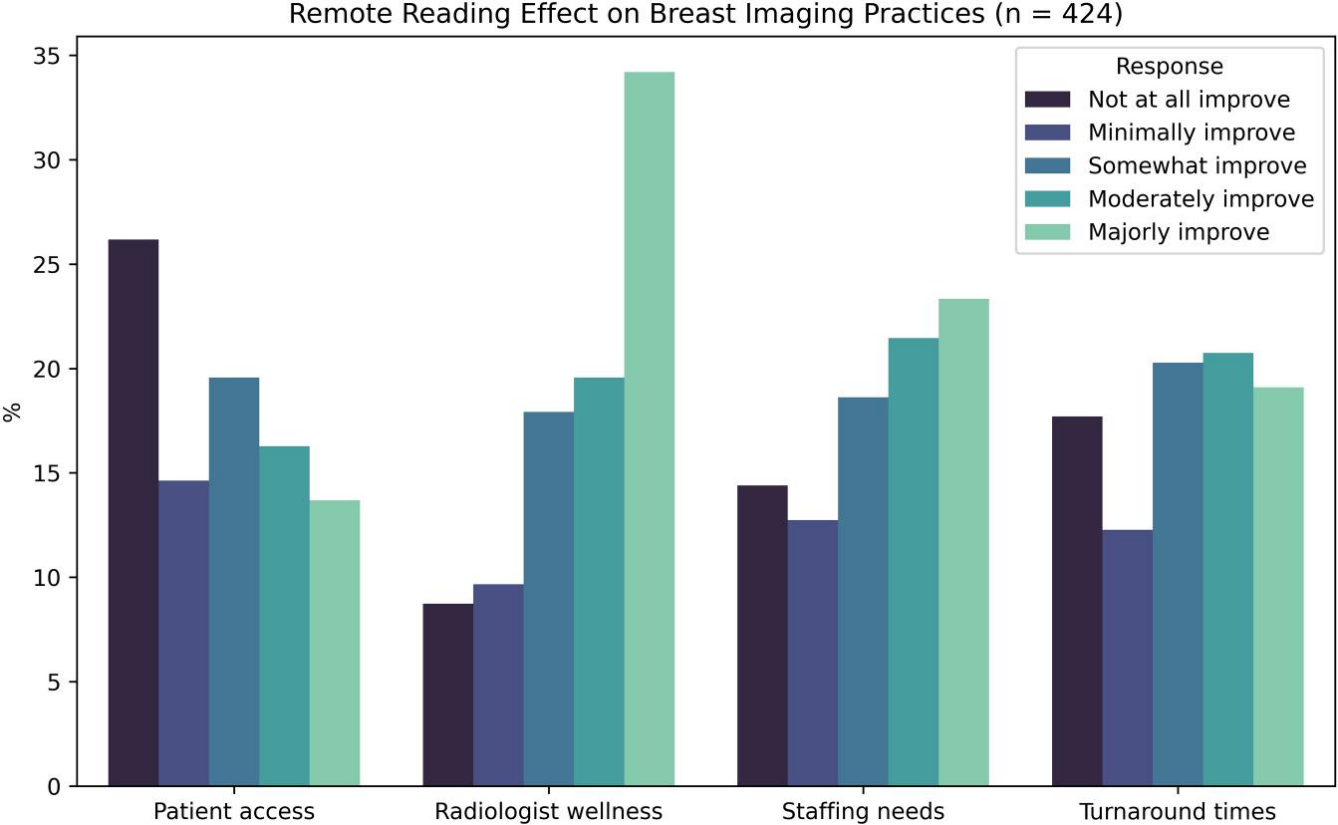
Responses to survey question 26: In your opinion, how would remote reading of breast imaging improve your practice? Responses based on 5-point Likert scale ranging from “not at all improve” to “majorly improve” (n = 424). Created from *SurveyMonkey*.^13^

Respondents were also asked to posit how remote reading of breast imaging could improve their practice. The most common responses that noted a major or moderate improvement were wellness (54%, 228/424), improved staffing (45%, 190/424), turnaround times (40%, 169/424), and patient access (33%, 127/424), as shown in Figure 3.

### Interest in remote reading

The majority of respondents (57%, 240/424) wished that their practice provided more breast remote reading opportunities, with 5% (22/424) indicating that they were already completely remote (Table 3). The desire for more remote reading opportunities was greatest among radiologists with dependents (68% [154/225] vs 50% [84/167]; *P* <.001) and younger radiologists (ages ≤40 years: 74% [62/84]; ages 41-50 years: 68% [74/109]; ages >50 years: 45%-56%; *P* = .001).

**Table 3.** Interest in Remote Reading

Questions	Answers	*N* = 424	%
Do you wish your practice provided more breast remote reading opportunities?	Yes	240	57
	No	84	20
	No preference	50	12
	Already completely remote	22	5
	Did not respond	28	7
What time of day would you be most interested in reading breast imaging remotely? Please check all that apply.	During the workday	312	74
	Early morning before typical workday	104	25
	Evenings after typical workday	109	26
	Weekends	144	34
	Did not respond	51	12
Can you see yourself working in a 100% remote reading practice in the future?	Yes	136	32
	No	215	51
	I don’t know	44	10
	Did not respond	29	7

Additionally, 32% (136/424) of respondents overall could see themselves working at a 100% remote practice in the future, which did not significantly differ based on participant or practice demographics.

### Job satisfaction and burnout

The validated question about job satisfaction indicated that 79% (333/424) of respondents were moderately to extremely satisfied with their jobs, 16% (66/424) were moderately to extremely dissatisfied with their jobs, 4% (17/424) were not sure, and 2% (8/424) did not respond (Table 4).

**Table 4.** Job Satisfaction and Burnout

		*N* = 424	%
Taking everything into consideration, how do you feel about your job as a whole?	I'm extremely dissatisfied	3	1
	I'm very dissatisfied	12	3
	I'm moderately dissatisfied	51	12
	I'm not sure	17	4
	I'm moderately satisfied	146	34
	I'm very satisfied	149	35
	I'm extremely satisfied	38	9
	Did not respond	8	2
Overall, based on your definition of burnout, how would you rate your level of burnout?	I enjoy my work, I have no symptoms of burnout	49	12
	Occasionally under stress and don't always have the energy, but I don't feel burned out	215	51
	I am definitely burning out and have symptoms of burnout, such as physical and emotional exhaustion	107	25
	Symptoms of burnout that I'm experiencing won't go away. I think about frustration at work a lot	33	8
	I feel completely burned out. I am the point where I may need some changes or to seek help	13	3
	Did not respond	7	2

The validated question about burnout indicated that 36% (153/424) of respondents reported feeling varying degrees of burnout with 25% (107/424) “definitely burning out,” 8% (33/424) experiencing persistent symptoms of burnout, and 3% (13/424) feeling “completely burned out.” Half of respondents (51%, 215/424) reported stress but no burnout (“occasionally under stress and don’t always have the energy, but I don’t feel burned out”).

Multiple-variable logistic regression analysis did not yield any significant relationship between reading breast imaging from home and job satisfaction (*P* = .301) or burnout (*P* = .140). In addition, no significant relationship between job satisfaction or burnout and any of the demographic variables was found in this multivariable model.

## Discussion

This study explored breast imaging radiologists’ views on remote reading, highlighting evolving job expectations. Key perceived benefits of remote reading included flexibility, efficiency, reduced commute time, extra income, and increased ability to care for dependents. The main drawback was limited patient contact, in particular the inability to personally perform breast US or examine patients. Most respondents wish for more remote reading options, and one-third envision a fully remote future. However, despite a strong preference for remote work and its perceived wellness benefits, there was no actual statistically significant association between having home workstations and improved job satisfaction or reduced burnout.

Remote reading was widely perceived to improve efficiency across age, gender, dependents, and practice types, with 58% of respondents rating it as a moderate or major advantage, compared with only 11% who felt it did not help. This perspective was even stronger than in a prior survey, wherein 46% of respondents reported greater efficiency reading breast studies remotely—possibly because of differences in question wording.^5^ However, perceived efficiency does not always equate to increased productivity, and studies on remote work’s impact on productivity are mixed. During the COVID-19 pandemic, remote work often led to decreased productivity, likely because of added family obligations, school closures, and other life stressors.^17^ Conversely, other studies have shown increased productivity in remote settings.^18,19^ Remote radiologists may be shielded from common workplace disruptions such as phone calls, nearby conversations, in-person consultations, contrast reaction coverage, and extended patient interactions.^20^ With fewer interruptions, they may complete tasks more quickly, but for this efficiency to translate into higher overall productivity, they must be assigned additional work. However, increased workloads could lead to greater burnout or decreased job satisfaction, potentially offsetting the well-being benefits of working from home. Departmental leadership must carefully balance these factors when considering remote work policies. Given declining radiology reimbursements, further research comparing remote and in-person productivity could help shape the future of this evolving practice.

By working remotely, breast imaging radiologists can often optimize their schedules to accommodate caregiving responsibilities, reduce travel time, and minimize interruptions. Notably, our study found that younger breast imaging radiologists sought more remote work options and were more likely to list work-life balance as a top motivation for remote work. These findings are in line with work trends across professions, which see Gen Z (born 1997-2012) and millennials (born 1981-1996) wanting more remote work options compared with Gen X (born 1965-1980) and baby boomers (born 1946-1964).^21,22^ As the breast imaging specialty evolves, it is essential to recognize and accommodate the growing preference among younger breast imaging radiologists for remote work, especially since most new entrants into the job market are Gen Z fellowship graduates. Creating a working environment that is attractive to younger breast imaging radiologists may also spur radiology residents to choose breast imaging as a subspecialty, which will help alleviate current and future workforce shortages.^23-25^

The top-ranked perceived drawbacks of remote breast imaging included the inability to perform US or examine patients, reduced patient contact, radiologist invisibility/commoditization, and IT issues. The inability to examine patients is specific to remote diagnostic breast imaging, and we previously reported that about half of breast imaging radiologists with home workstations read diagnostic mammograms or US remotely.^10^ In the free-response sections of the survey, breast imaging radiologists cited challenges such as relying on technologists for physical examination findings or unclear US and reduced patient and technologist rapport. However, they also emphasized the necessity of remote diagnostics to provide access for underserved populations, particularly amid a breast radiologist shortage.^4^ Most agreed that, with advanced technology, well-trained staff, and strong remote communication, remote diagnostic programs could still deliver exceptional patient care.

The shift toward remote radiology also raises concerns about its impact on training and professional development. A critical component of breast imaging is the continuous feedback loop between reading screening cases, performing diagnostic examinations, and conducting biopsies while assessing radiology–pathology correlations. This iterative process is particularly valuable for less experienced radiologists because it contributes to continuous professional growth.^26^ Unfortunately, this feedback loop is diminished in remote radiology, which may impact the development of radiological expertise. One academic institution reported that 81% of first- and second-year radiology residents felt remote work during the pandemic had a negative impact on their training.^27^ Ensuring that remote radiology does not hinder professional growth will require innovative solutions, such as structured mentorship, enhanced virtual collaboration, and deliberate efforts to maintain the critical feedback loop essential for skill development.

Another potential drawback of remote work is its impact on the invisibility and potential commoditization of breast imaging radiologists. As noted above, more than half of breast imaging radiologists expressed a desire for increased remote reading opportunities, with one-third envisioning themselves reading remotely 100% of the time. As radiologists become less physically present in clinical settings, their role may be perceived as more transactional, reducing opportunities for direct collaboration with providers, technologists, and patients.^28^ Additionally, as artificial intelligence (AI) continues to advance, there is a growing concern that radiologists working in remote, high-volume environments could be viewed as interchangeable service providers rather than specialized experts offering nuanced, patient-centered interpretations.^29,30^ While virtual health care has become more accepted, radiologists must actively find ways to reinforce their relevance, whether through virtual patient consultations, increased engagement with multidisciplinary teams, or advocacy for their expertise in AI integration.^31^ By proactively defining their evolving role, radiologists can ensure that remote work enhances—rather than diminishes—their impact on patient care.

Survey responses indicate that, while breast imaging radiologists believe remote work would improve wellness, data show otherwise: Those with mammography home workstations reported similar job satisfaction and burnout levels as those without, refuting our hypothesis. It is possible that radiologists with lower baseline wellness have already selectively chosen to work remotely to improve their wellness to the levels of the larger community. In addition, remote reading is a relatively new practice, and perhaps more time is needed to capture its wellness benefit. Nonetheless, this finding highlights a critical insight: Perceived benefits of remote work may not align with actual benefits. While reduced commuting and increased flexibility can relieve stress, in-person interactions with colleagues and patients often drive job satisfaction, and social isolation or lack of work-life boundaries may elevate burnout.^17,32,33^ It is also possible that the positive aspects of remote reading are offset by the stress of performing remote diagnostic examinations. For practices implementing remote or hybrid models, strategies to retain in-person benefits—like social connections and face-to-face patient interactions—are essential. Remote reading from different geographic locations, and especially different time zones, may also add challenges.

This study has several limitations. First, it targeted SBI members, who may be more engaged than the general population of breast imaging radiologists. As a result, the findings might not fully reflect all breast imaging radiologists’ views on remote breast imaging, especially among community-based general breast imaging radiologists. Additionally, SBI members with strong opinions may have been more likely to respond, potentially skewing the results. Regarding the burnout findings, it is possible that individuals working remotely would experience greater burnout if required to work on-site, while some on-site radiologists might face higher burnout if working remotely. This self-selection of work arrangements may explain the lack of a significant difference observed between the 2 groups. Our response rate was relatively low at 13% (424/3244), limiting generalizability. In addition, we did not evaluate the effects of varying work conditions at home, such as having a dedicated reading room vs a shared space, being alone vs having others present (eg, small children requiring attention), or differences in case volume and complexity between remote and in-office settings. This cross-sectional study provides a snapshot of 2023; opinions on remote reading may evolve with technological advancements and societal changes. Finally, our evaluations of job satisfaction and burnout were each based on single-item validated questions, which may yield different findings than more extensive tools. In particular, the single-item question on burnout is useful for comparing burnout rates within a single population. However, it is not valid for historical trends in comparing with previous studies in breast imaging done with the full Maslach Burnout Inventory.^34^

## Conclusion

Most breast imaging radiologists, especially younger ones, seek more remote work opportunities, valuing flexibility, efficiency, and work-life balance. However, this interest must be weighed against drawbacks such as reduced patient contact and potential radiologist invisibility. Notably, despite the perception that remote work enhances wellness, breast imaging radiologists with home workstations reported similar job satisfaction and burnout levels to those without home workstations. Remote breast imaging is a complex issue, requiring further research to better understand its benefits and challenges for both radiologists and patients and to develop strategies to optimize benefits while minimizing downsides.

## Declaration of generative AI and AI-assisted technologies in the writing process

During the preparation of this work, the authors used ChatGPT in order to improve the grammar and clarity of portions of the Introduction and Discussion sections. After using this tool/service, the authors reviewed and edited the content as needed and take full responsibility for the content of the publication.

## Supplementary Material

wbaf039_Supplementary_Data
