# Supplementary material for: Breast Remote Reading: Widely Desired But Home Workstations Show No Association With Job Satisfaction Or Burnout
Source: J Breast Imaging. 2025 Nov 3;7(6):685–95. doi: 10.1093/jbi/wbaf039 (PMC12700671; doi:10.1093/jbi/wbaf039)
Supplement: wbaf039_Supplementary_Data [file wbaf039_supplementary_data.docx]

**APPENDIX A**

**SBI Patient Care and Delivery Committee Survey: Home Workstations and Remote Diagnostic**

**Breast Imaging**

*Demographics*

1. What is your age?
   1. (textbox for free response)
2. What is your gender identity?
   1. Woman
   2. Man
   3. Transgender
   4. Non-binary/non-conforming
   5. Prefer not to answer
   6. Other ___
3. What is your race/ethnicity? Please check all that apply.
   1. White
   2. Black or African American
   3. Hispanic, Latino, or Spanish Origin
   4. American Indian or Alaska Native
   5. Asian or Asian American
   6. Native Hawaiian or other Pacific Islander
   7. Prefer not to answer
   8. Other ____
4. How many years have you been in practice after training? **Please select “0” if you’re a trainee.**
   1. (textbox for free response)
5. What type of practice do you work in?
   1. Academic
   2. Private
   3. Academic/private hybrid (i.e. community practice affiliated with academic medical center)
   4. Military/VA
   5. Tele-radiology
   6. Other ____
6. Do you have an official leadership role in your practice?
   1. Yes
   2. No
7. In what state do you practice?
   1. (drop-down list of states)
8. Do you have dependents (e.g. children, elderly) at home?
   1. Yes
   2. No
9. Taking everything into consideration, how do you feel about your job as a whole?
   1. I’m extremely dissatisfied
   2. I’m very dissatisfied
   3. I’m moderately satisfied
   4. I’m not sure
   5. I’m very satisfied
   6. I’m extremely satisfied
10. Overall, based on your definition of burnout, how would you rate your level of burnout?
    1. I enjoy my work, I have no symptoms of burnout
    2. Occasionally under stress and don't always have the energy, but I don't feel burned out
    3. I am definitely burning out and have symptoms of burnout, such as physical and emotional exhaustion
    4. Symptoms of burnout that I'm experiencing won't go away. I think about frustration at work a lot
    5. I feel completely burned out. I am the point where I may need some changes or to seek help

*Home/Personal Remote Workstations*

1. What percentage of your clinical time is breast imaging?
   1. (textbox for free response)
2. Do you read breast imaging exams remotely from home or other personal remote site (e.g. remote office space) as part of your routine clinical practice?
   1. Yes
   2. No
3. What percentage of your breast clinical time do you read from home or at a personal remote site?
   1. (textbox for free response)
4. Which of the following types of studies are you reading from a home or personal remote workstation? Please check all that apply.
   1. Screening Mammography (FFDM or DBT)
   2. Screening Ultrasound
   3. Diagnostic Mammography (FFDM or DBT)
   4. Diagnostic Ultrasound
   5. MRI - diagnostic or screening
   6. Contrast Enhanced Digital Mammography
   7. Molecular Breast Imaging (MBI or PEM)
5. What time of day do you primarily read breast imaging from a home or personal remote workstation? Please check all that apply.
   1. During the workday
   2. Early morning before typical workday
   3. Evenings after typical workday
   4. Weekends
6. What are your top three motivations for reading from home or a personal remote site?
   1. Personal health issue
   2. Decrease time commuting
   3. Dependent care (children, elderly, etc.)
   4. Personal preference working solo
   5. Flexibility/work-life balance
   6. Extra income
   7. Limit time spent discussing results/recommendations/procedures with patients
   8. Limit challenging patient interactions
   9. Other ____
7. How does working from home or a personal remote site affect your efficiency?
   1. Much less efficient
   2. Less efficient
   3. No change
   4. More efficient
   5. Much more efficient

*Perceptions about Breast Remote Reading*

**Definition: the term “Breast Remote Reading” encompasses all types of remote breast imaging, including reading exams from home and/or performing remote diagnostic visits either from home or a central/local site.**

1. Do you wish your practice provided more breast remote reading opportunities?
   1. Yes
   2. No
   3. No preference
   4. Already completely remote
2. What time of day would you be most interested in reading breast imaging remotely? Please check all that apply.
   1. During the workday
   2. Early morning before typical workday
   3. Evenings after typical workday
   4. Weekends
3. What do you see as the primary advantages of breast remote reading?

(options to rank each of the following advantages as: not an advantage, minimal advantage, somewhat of an advantage, moderate advantage, or major advantage)

- 1. Patient access
  2. COVID safety
  3. Flexibility/work-life balance
  4. Efficiency
  5. Salary
  6. Relieve volume for on-site staff
  7. Other ___

1. What do you see as the drawbacks of breast remote reading?

(options to rank each of the following drawbacks as: not an advantage, minimal advantage, somewhat of an advantage, moderate advantage, or major advantage)

- 1. Cost of equipment
  2. Equipment maintenance / IT issues
  3. Space at home
  4. Impaired patient contact / connection
  5. Social isolation / Impaired colleague interaction / Collaboration
  6. Decreased teaching / "face time" with trainees
  7. Academic productivity and promotion opportunities compromised
  8. Radiologist unable to scan or examine patient when needed (e.g. for diagnostic ultrasounds)
  9. Poor accessibility to radiologists for clinicians
  10. Invisibility and commoditization of radiologists
  11. Other ___

1. How likely do you think breast remote reading will be a major practice pattern in the future?
   1. Very likely
   2. Likely
   3. Neutral
   4. Unlikely
   5. Very unlikely
2. Can you see yourself working in a 100% remote reading practice in the future?
   1. Yes
   2. No
   3. I don’t know

*Future Practice Goals for Breast Remote Reading*

1. Does ***your practice*** plan to increase breast imaging remote reading options for radiologists?
   1. Yes
   2. No
   3. I don’t know
   4. N/a already 100% remote
2. If ***your practice*** plans to increase breast remote reading, what is the anticipated timeline?
   1. Within a year
   2. 1-2 years
   3. 3-5 years
   4. >5 years
   5. I don't know
   6. N/A, no plans
3. In your opinion, how would remote reading of breast imaging improve ***your practice***?

(options to rank each of the following choices as: not at all improve, minimally improve, somewhat improve, moderately improve, or majorly improve).

- 1. Patient access
  2. Turnaround times
  3. Staffing needs
  4. Radiologist wellness
  5. Other ___

**Thank you for completing the primary component of this survey.**

**For those of you who have experience or knowledge regarding *remote DIAGNOSTIC breast imaging*, please continue with 15 additional *optional* questions that will provide insight into this emerging aspect of our clinical practice. We would be enormously grateful if you could take an additional 5-8 minutes to complete them.**

**Definition of Remote Diagnostic Breast Imaging: Interpreting *diagnostic* mammograms and breast ultrasounds remotely (either from home or a central/local site) and communicating with the technologists and the patients through some virtual platform.**

1. Do you have experience or knowledge regarding remote diagnostic breast imaging?
   1. Yes, take me to the Remote Diagnostic Breast Imaging survey questions
   2. No, complete the survey here
2. For **remote diagnostic exams**, how does the radiologist typically communicate results to the patient? **Please select all that apply**.
   1. Phone
   2. Video consultation
   3. Surrogate speaks to patient in-person (trainee, technologist, radiology assistant, etc.)
   4. Communicate via the referring clinician
   5. Other (please specify) ____
3. For **remote diagnostic exams**, how does the radiologist typically communicate with the technologists?
   1. Phone
   2. Video chat (e.g. zoom, etc.)
   3. Text chat (e.g. jabber, etc.)
   4. Other (please specify) ____
4. Does your practice have any **exclusion criteria** for **remote diagnostic exams**? I.e. types of visits that **are not** permitted to be done remotely? Please check all that apply.
   1. None, all types of diagnostics can be done remotely
   2. Post-lumpectomy
   3. Focal pain
   4. Diffuse pain
   5. Palpable
   6. Nipple discharge
   7. Skin changes
   8. Callback from screening
   9. Cancer Staging
   10. Response to neoadjuvant
   11. Axillary cases
   12. I don't know
   13. Other (please specify) ____
5. How is ultrasound performed for **remote diagnostic exams**?
   1. Sonographer on site
   2. Automated breast ultrasound
   3. Trainee performs ultrasound
   4. Other (please specify) ____
6. Has your practice had any ethical/legal issues arise with **remote diagnostic exams**?
   1. Yes
   2. No
   3. I don’t know
   4. Other ___
7. Has your practice had any verbal or written patient complaints related to **remote diagnostic exams**?
   1. Yes
   2. No
   3. I don’t know
   4. Other ___
8. Has your practice received any positive feedback from patients regarding **remote diagnostic exams**?
   1. Yes
   2. No
   3. I don’t know
   4. Other ___
9. How do you perceive patient acceptance of **remote diagnostic exams**?
   1. Patients much prefer remote option
   2. Patients slightly prefer remote option
   3. Patients like in-person and remote option equally
   4. Patients slightly prefer in-person
   5. Patients much prefer in-person
   6. Patients are generally unaware that an in-person non-remote alternative exists
   7. Other (please specify) ____
10. Do you feel you are able to establish the same level of rapport/connection with patients during **remote diagnostic exams** compared to in-person? In particular when delivering ***bad news***?
    1. Much less effective
    2. Less effective
    3. Equally effective/same
    4. More effective
    5. Much more effective
11. Do you feel you are able to establish the same level of rapport and trust with technologists during **remote diagnostic exams** compared to in-person?
    1. Much less effective
    2. Less effective
    3. Equally effective/same
    4. More effective
    5. Much more effective
12. Has your practice added staffing to support **remote diagnostic exams**, such as nurse navigators or patient coordinators?
    1. Yes
    2. No
    3. Other (please specify) ___
13. How does your practice handle physical exam findings (e.g. for skin changes, nipple discharge, palpable) for **remote diagnostic exams**?
    1. (textbox for free response)
14. How does your practice handle repeat ultrasound scanning by the radiologist (e.g. when radiologist wants to scan realtime to troubleshoot etc.) for **remote diagnostic exams**?
    1. (textbox for free response)
15. Please provide strategies for building rapport with patients remotely.
    1. (textbox for free response)
16. Please provide strategies for building rapport with technologists remotely.
    1. (textbox for free response)
17. Please provide any additional thoughts here.
    1. (textbox for free response)

**Appendix B. Differences in responses by gender**

|  |  | **Men (%)** | **Women (%)** | **p-value** |
| --- | --- | --- | --- | --- |
| **What are your top three motivations for reading from home or a personal remote site? *** | *Personal health issue* | 0/25 (0) | 6/101 (6) | 0.469 |
|  | *Decrease time commuting* | 15/25 (60) | 52/101 (51) | 0.589 |
|  | *Dependent care (children, elderly, etc.)* | 1/25 (4) | 28/101 (28) | **0.024** |
|  | *Personal preference working solo* | 2/25 (8) | 11/101 (11) | 0.954 |
|  | *Flexibility/work-life balance* | 15/25 (60) | 71/101 (70) | 0.453 |
|  | *Extra income* | 12/25 (48) | 20/101 (20) | **0.008** |
|  | *Limit time spent discussing results/procedures with patients* | 0/25 (0) | 4/101 (4) | 0.708 |
|  | *Limit challenging patient interactions* | 1/25 (4) | 2/101 (2) | 1 |
|  | *Did not respond* | 1 | 2 | – |
| **How does working from home or a personal remote site affect your efficiency?** | *Much less efficient* | 1/25 (4) | 1/101 (1) | 0.854 |
|  | *Less efficient* | 3/25 (12) | 13/101 (13) | 1 |
|  | *No change* | 5/25 (20) | 18/101 (18) | 1 |
|  | *More efficient* | 12/25 (48) | 31/101 (31) | 0.162 |
|  | *Much more efficient* | 4/25 (16) | 38/101 (38) | 0.069 |
|  | *Did not respond* | 1 | 2 | – |
| **Do you wish your practice provided more breast remote reading opportunities?** | *Yes* | 57/91 (63) | 175/290 (60) | 0.789 |
|  | *No* | 21/91 (23) | 58/290 (20) | 0.629 |
|  | *No preference* | 11/91 (12) | 38/290 (13) | 0.942 |
|  | *Already completely remote* | 2/91 (2) | 19/290 (7) | 0.185 |
|  | *Did not respond* | 7 | 19 | – |
| **What time of day would you be most interested in reading breast imaging remotely? Please check all that apply.** | *During the workday* | 68/86 (79) | 233/274 (85) | 0.255 |
|  | *Early morning before typical workday* | 20/86 (23) | 81/274 (30) | 0.318 |
|  | *Evenings after typical workday* | 30/86 (35) | 75/274 (27) | 0.23 |
|  | *Weekends* | 29/86 (34) | 109/274 (40) | 0.378 |
|  | *Did not respond* | 12 | 25 | – |
| **Can you see yourself working in a 100% remote reading practice in the future?** | *Yes* | 32/90 (36) | 98/290 (34) | 0.857 |
|  | *No* | 49/90 (54) | 158/290 (54) | 1 |
|  | *I don't know* | 9/90 (10) | 34/290 (12) | 0.794 |
|  | *Did not respond* | 8 | 19 | – |

Comparison of proportions (%) using Pearson chi-square test. Respondents were excluded from analysis if they did not respond to the question. *Throughout the survey, “Reading from home” was further defined to include reading from home *or* other personal remote site.

**Appendix C. Differences in responses by age group**

|  |  | **≤ 40**  **(%)** | **41-50 (%)** | **51-60 (%)** | **61-70 (%)** | **> 70 (%)** | **p-value** |
| --- | --- | --- | --- | --- | --- | --- | --- |
| **What are your top three motivations for reading from home or a personal remote site? *** | *Personal health issue* | 0/18(0) | 2/38 (5) | 1/39 (3) | 1/24 (4) | 2/9 (22) | 0.111 |
|  | *Decrease time commuting* | 12/18 (67) | 20/38 (53) | 20/39 (51) | 10/24 (42) | 4/9 (44) | 0.594 |
|  | *Dependent care (children, elderly, etc.)* | 13/18 (72) | 9/38 (24) | 5/39 (13) | 1/24 (4) | 0/9 (0) | **<0.001** |
|  | *Personal preference working solo* | 1/18 (6) | 5/38 (13) | 3/39 (8) | 2/24 (8) | 2/9 (22) | 0.631 |
|  | *Flexibility/work-life balance* | 16/18 (89) | 30/38 (79) | 26/39 (67) | 12/24 (50) | 2/9 (22) | **0.001** |
|  | *Extra income* | 2/18 (11) | 11/38 (29) | 14/39 (36) | 5/24 (21) | 0/9 (0) | 0.097 |
|  | *Limit time spent discussing results/procedures with patients* | 1/18 (6) | 2/38 (5) | 1/39 (3) | 1/24 (4) | 0/9 (0) | 0.928 |
|  | *Limit challenging patient interactions* | 1/18 (6) | 0/38 (0) | 0/39 (0) | 1/24 (4) | 1/9 (11) | 0.197 |
|  | *Did not respond* | 0 | 3 | 0 | 1 | 0 | – |
| **How does working from home or a personal remote site affect your efficiency?** | *Much less efficient* | 0/18 (0) | 0/38 (0) | 1/39 (3) | 0/24 (0) | 1/9 (11) | 0.144 |
|  | *Less efficient* | 2/18 (11) | 5/38 (13) | 5/39 (13) | 4/24 (17) | 0/9 (0) | 0.788 |
|  | *No change* | 4/18 (22) | 12/38 (32) | 6/39 (15) | 1/24 (4) | 2/9 (22) | 0.105 |
|  | *More efficient* | 4/18 (22) | 10/38 (26) | 15/39 (38) | 12/24 (50) | 2/9 (22) | 0.216 |
|  | *Much more efficient* | 8/18 (44) | 11/38 (29) | 12/39 (31) | 7/24 (29) | 4/9 (44) | 0.711 |
|  | *Did not respond* | 0 | 3 | 0 | 1 | 0 | – |
| **Do you wish your practice provided more breast remote reading opportunities?** | *Yes* | 62/84 (74) | 74/109 (68) | 62/110 (56) | 30/67 (45) | 10/22 (45) | **0.001** |
|  | *No* | 10/84 (12) | 14/109 (13) | 29/110 (26) | 21/67 (31) | 8/22 (36) | **0.001** |
|  | *No preference* | 8/84 (10) | 13/109 (12) | 13/110 (12) | 13/67 (19) | 3/22 (14) | 0.46 |
|  | *Already completely remote* | 4/84 (5) | 8/109 (7) | 6/110 (5) | 3/67 (4) | 1/22 (5) | 0.918 |
|  | *Did not respond* | 4 | 9 | 5 | 7 | 3 | – |
| **What time of day would you be most interested in reading breast imaging remotely? Please check all that apply.** | *During the workday* | 71/82 (87) | 90/104 (87) | 76/101 (75) | 55/63 (87) | 17/20 (85) | 0.138 |
|  | *Early morning before typical workday* | 30/82 (37) | 28/104 (27) | 32/101 (32) | 12/63 (19) | 2/20 (10) | 0.051 |
|  | *Evenings after typical workday* | 34/82 (41) | 33/104 (32) | 27/101 (27) | 10/63 (16) | 4/20 (20) | **0.012** |
|  | *Weekends* | 35/82 (43) | 39/104 (38) | 43/101 (43) | 19/63 (30) | 7/20 (35) | 0.508 |
|  | *Did not respond* | 6 | 14 | 14 | 11 | 5 | – |
| **Can you see yourself working in a 100% remote reading practice in the future?** | *Yes* | 33/84 (39) | 44/109 (40) | 29/110 (26) | 22/67 (33) | 7/21 (33) | 0.212 |
|  | *No* | 42/84 (50) | 55/109 (50) | 70/110 (64) | 36/67 (54) | 9/21 (43) | 0.181 |
|  | *I don't know* | 9/84 (11) | 10/109 (9) | 11/110 (10) | 9/67 (13) | 5/21 (24) | 0.367 |
|  | *Did not respond* | 4 | 9 | 5 | 7 | 4 | – |

Comparison of proportions (%) using Pearson chi-square test. *P*-values in bold are statistically significant. Respondents were excluded from analysis if they did not respond to the question.

*Throughout the survey, “Reading from home” was further defined to include reading from home *or* other personal remote site.

**Appendix D. Differences in responses based on presence of dependents at home**

|  |  | **No dependent (%)** | **Has dependent (%)** | **p-value** |
| --- | --- | --- | --- | --- |
| **What are your top three motivations for reading from home or a personal remote site? *** | *Personal health issue* | 3/50 (6) | 3/78 (4) | 0.893 |
|  | *Decrease time commuting* | 27/50 (54) | 39/78 (50) | 0.794 |
|  | *Dependent care (children, elderly, etc.)* | 1/50 (2) | 27/78 (35) | **<0.001** |
|  | *Personal preference working solo* | 5/50 (10) | 8/78 (10) | 1 |
|  | *Flexibility/work-life balance* | 27/50 (54) | 59/78 (76) | **0.019** |
|  | *Extra income* | 9/50 (18) | 23/78 (29) | 0.209 |
|  | *Limit time spent discussing results/procedures with patients* | 2/50 (4) | 3/78 (4) | 1 |
|  | *Limit challenging patient interactions* | 2/50 (4) | 1/78 (1) | 0.694 |
|  | *Did not respond* | 2 | 2 | – |
| **How does working from home or a personal remote site affect your efficiency?** | *Much less efficient* | 2/50 (4) | 0/78 (0) | 0.294 |
|  | *Less efficient* | 3/50 (6) | 13/78 (17) | 0.132 |
|  | *No change* | 7/50 (14) | 18/78 (23) | 0.301 |
|  | *More efficient* | 22/50 (44) | 21/78 (27) | 0.071 |
|  | *Much more efficient* | 16/50 (32) | 26/78 (33) | 1 |
|  | *Did not respond* | 2 | 2 | – |
| **Do you wish your practice provided more breast remote reading opportunities?** | *Yes* | 84/167 (50) | 154/225 (68) | **<0.001** |
|  | *No* | 47/167 (28) | 35/225 (16) | **0.004** |
|  | *No preference* | 29/167 (17) | 21/225 (9) | **0.028** |
|  | *Already completely remote* | 7/167 (4) | 15/225 (7) | 0.406 |
|  | *Did not respond* | 11 | 15 | – |
| **What time of day would you be most interested in reading breast imaging remotely? Please check all that apply.** | *During the workday* | 133/154 (86) | 176/216 (81) | 0.269 |
|  | *Early morning before typical workday* | 32/154 (21) | 72/216 (33) | **0.011** |
|  | *Evenings after typical workday* | 39/154 (25) | 69/216 (32) | 0.206 |
|  | *Weekends* | 49/154 (32) | 94/216 (44) | **0.030** |
|  | *Did not respond* | 24 | 24 | – |
| **Can you see yourself working in a 100% remote reading practice in the future?** | *Yes* | 56/166 (34) | 79/225 (35) | 0.861 |
|  | *No* | 90/166 (54) | 122/225 (54) | 1 |
|  | *I don't know* | 20/166 (12) | 24/225 (11) | 0.791 |
|  | *Did not respond* | 12 | 15 | – |

Comparison of proportions (%) using Pearson chi-square test. Respondents were excluded from analysis if they did not respond to the question.

*Throughout the survey, “Reading from home” was further defined to include reading from home *or* other personal remote site.

**Appendix E. Differences in responses by geographic region**

|  |  | **MW (%)** | **NE (%)** | **S (%)** | **SW (%)** | **W (%)** | **p-value** |
| --- | --- | --- | --- | --- | --- | --- | --- |
| **What are your top three motivations for reading from home or a personal remote site? *** | *Personal health issue* | 1/16 (6) | 1/42 (2) | 0/32 (0) | 2/20 (10) | 1/12 (8) | 0.381 |
|  | *Decrease time commuting* | 8/16 (50) | 24/42 (57) | 18/32 (56) | 10/20 (50) | 5/12 (42) | 0.883 |
|  | *Dependent care (children, elderly, etc.)* | 5/16 (31) | 9/42 (21) | 7/32 (22) | 7/20 (35) | 0/12 (0) | 0.206 |
|  | *Personal preference working solo* | 2/16 (12) | 4/42 (10) | 3/32 (9) | 3/20 (15) | 0/12 (0) | 0.726 |
|  | *Flexibility/work-life balance* | 8/16 (50) | 30/42 (71) | 22/32 (69) | 13/20 (65) | 7/12 (58) | 0.595 |
|  | *Extra income* | 2/16 (13) | 11/42 (26) | 8/32 (25) | 5/20 (25) | 6/12 (50) | 0.277 |
|  | *Limit time spent discussing results/procedures with patients* | 0/16 (0) | 1/42 (2) | 1/32 (3) | 0/20 (0) | 2/12 (17) | 0.088 |
|  | *Limit challenging patient interactions* | 0/16 (0) | 0/42 (0) | 2/32 (6) | 0/20 (0) | 1/12 (8) | 0.23 |
|  | *Did not respond* | 2 | 0 | 1 | 1 | 0 | – |
| **How does working from home or a personal remote site affect your efficiency?** | *Much less efficient* | 1/16 (6) | 1/42 (2) | 0/32 (0) | 0/20 (0) | 0/12 (0) | 0.506 |
|  | *Less efficient* | 3/16 (19) | 2/42 (5) | 3/32 (9) | 3/20 (15) | 3/12 (25) | 0.263 |
|  | *No change* | 3/16 (19) | 9/42 (21) | 7/32 (22) | 4/20 (20) | 2/12 (17) | 0.995 |
|  | *More efficient* | 5/16 (31) | 15/42 (36) | 12/32 (38) | 7/20 (35) | 3/12 (25) | 0.95 |
|  | *Much more efficient* | 4/16 (25) | 15/42 (36) | 10/32 (31) | 6/20 (30) | 4/12 (33) | 0.954 |
|  | *Did not respond* | 2 | 0 | 1 | 1 | 0 | – |
| **Do you wish your practice provided more breast remote reading opportunities?** | *Yes* | 34/56 (61) | 61/107 (57) | 68/104(65) | 22/45 (49) | 41/64 (64) | 0.352 |
|  | *No* | 11/56 (20) | 24/107 (22) | 21/104 (20) | 12/45 (27) | 12/64 (19) | 0.866 |
|  | *No preference* | 8/56 (14) | 15/107 (14) | 11/104 (11) | 6/45 (13) | 9/64 (14) | 0.94 |
|  | *Already completely remote* | 3/56 (5) | 7/107 (7) | 4/104 (4) | 5/45 (11) | 2/64 (3) | 0.389 |
|  | *Did not respond* | 8 | 4 | 5 | 4 | 4 | – |
| **What time of day would you be most interested in reading breast imaging remotely? Please check all that apply.** | *During the workday* | 46/53 (87) | 90/103 (87) | 81/96 (84) | 36/42 (86) | 47/61 (77) | 0.478 |
|  | *Early morning before typical workday* | 15/53 (28) | 26/103 (25) | 30/96 (31) | 11/42 (26) | 15/61 (25) | 0.869 |
|  | *Evenings after typical workday* | 17/53 (32) | 27/103 (26) | 28/96 (29) | 9/42 (21) | 21/61 (34) | 0.613 |
|  | *Weekends* | 20/53 (38) | 38/103 (37) | 39/96 (41) | 13/42 (31) | 26/61 (43) | 0.781 |
|  | *Did not respond* | 11 | 8 | 13 | 7 | 7 | – |
| **Can you see yourself working in a 100% remote reading practice in the future?** | *Yes* | 19/56 (34) | 32/107 (30) | 44/104 (42) | 16/45 (36) | 17/63 (27) | 0.247 |
|  | *No* | 33/56 (59) | 62/107 (58) | 48/104 (46) | 25/45 (56) | 36/63 (57) | 0.397 |
|  | *I don't know* | 4/56 (7) | 13/107 (12) | 12/104 (12) | 4/45 (9) | 10/63 (16) | 0.63 |
|  | *Did not respond* | 8 | 4 | 5 | 4 | 5 | – |

Comparison of proportions (%) using Pearson chi-square test. *P*-values in bold are statistically significant. Respondents were excluded from analysis if they did not respond to the question. Abbreviations: MW, Midwest; NE, Northeast; S, South; SW, Southwest; W, West. *Throughout the survey, “Reading from home” was further defined to include reading from home *or* other personal remote site.

**Appendix F. Differences in responses by practice type**

|  |  | **Academic* (%)** | **Private (%)** | **p-value** |
| --- | --- | --- | --- | --- |
| **What are your top three motivations for reading from home or a personal remote site?**** | *Personal health issue* | 1/53 (2) | 1/53 (2) | 1 |
|  | *Decrease time commuting* | 31/53 (58) | 25/53 (47) | 0.331 |
|  | *Dependent care (children, elderly, etc.)* | 11/53 (21) | 10/53 (19) | 1 |
|  | *Personal preference working solo* | 5/53 (9) | 4/53 (8) | 1 |
|  | *Flexibility/work-life balance* | 39/53 (74) | 29/53 (55) | 0.068 |
|  | *Extra income* | 13/53 (25) | 12/53 (23) | 1 |
|  | *Limit time spent discussing results/procedures with patients* | 0/53 (0) | 4/53 (8) | 0.126 |
|  | *Limit challenging patient interactions* | 1/53 (2) | 1/53 (2) | 1 |
|  | *Did not respond* | 0 | 3 | – |
| **How does working from home or a personal remote site affect your efficiency?** | *Much less efficient* | 1/53 (2) | 0/53 (0) | 1 |
|  | *Less efficient* | 5/53 (9) | 8/53 (15) | 0.554 |
|  | *No change* | 11/53 (21) | 11/53 (21) | 1 |
|  | *More efficient* | 17/53 (32) | 21/53 (40) | 0.543 |
|  | *Much more efficient* | 19/53 (36) | 13/53 (25) | 0.29 |
|  | *Did not respond* | 0 | 3 | – |
| **Do you wish your practice provided more breast remote reading opportunities?** | *Yes* | 121/185 (65) | 106/177 (60) | 0.329 |
|  | *No* | 35/185 (19) | 44/177 (25) | 0.215 |
|  | *No preference* | 25/185 (14) | 21/177 (12) | 0.754 |
|  | *Already completely remote* | 4/185 (2) | 6/177 (3) | 0.695 |
|  | *Did not respond* | 10 | 14 | – |
| **What time of day would you be most interested in reading breast imaging remotely? Please check all that apply.** | *During the workday* | 153/180 (85) | 131/161 (81) | 0.452 |
|  | *Early morning before typical workday* | 59/180 (33) | 39/161 (24) | 0.105 |
|  | *Evenings after typical workday* | 62/180 (34) | 37/161 (23) | **0.027** |
|  | *Weekends* | 76/180 (42) | 55/161 (34) | 0.157 |
|  | *Did not respond* | 15 | 30 | – |
| **Can you see yourself working in a 100% remote reading practice in the future?** | *Yes* | 51/185 (28) | 63/176 (36) | 0.117 |
|  | *No* | 115/185 (62) | 89/176 (51) | **0.034** |
|  | *I don't know* | 19/185 (10) | 24/176 (14) | 0.41 |
|  | *Did not respond* | 10 | 15 | – |

Comparison of proportions (%) using Pearson chi-square test. *P*-values in bold are statistically significant. Respondents were excluded from analysis if their indicated practice type was military/VA, teleradiology, other, or they did not report their practice type. *Combination of academic and academic/private hybrid. **Throughout the survey, “Reading from home” was further defined to include reading from home *or* other personal remote site.
